# Association of Electronic Media Use and Sleep Habits Among Secondary School Students in Al-Madinah

**DOI:** 10.7759/cureus.22334

**Published:** 2022-02-17

**Authors:** Nojood Saud Al-Anazi, Zainab Al-Harbi

**Affiliations:** 1 Family Medicine, Academy of Family Medicine, Al-Madinah, SAU

**Keywords:** mobile phone addiction, madinah, school children., electronic media use, sleep pattern

## Abstract

Objectives: To ascertain the relationship between electronic media use and sleep patterns among secondary school students in Al-Madinah, Saudi Arabia.

Methods: This was a descriptive cross-sectional study, conducted from July 2021 to December 2021. This study examined eight female secondary high schools, in Al-Madinah, Saudi Arabia. The sampling technique used was the stratification of governmental schools according to their location in the north, south, east, and west. According to the WHO sample size calculator, the sample size was 375. The Statistical Package for the Social Sciences version 26 (SPSS version 26) (SPSS Incl., Chicago, IL) was used for the analysis of data.

Results: A total of 388 female students were recruited, from the age group 12 to 40 years, with a mean age group of 16.45 ± 4.25 years. The majority of participants in our research utilized various types of electronic devices before going to bed. Some 335 individuals reported using electronic devices on a daily basis while at school, while 357 reported using electronic devices prior to sleeping on weekends. The more time spent on electronic gadgets, the more sleep is interrupted (p-value = 0.005). This condition was more prevalent among older children: 55% of third-year children and 41% of second-year children reported having it (p = 0.01). More than 20% had difficulty sleeping. Around 5% of respondents reported experiencing frequent nightly awakenings, whereas 23% reported feeling drowsy/sleepy feeling at school. After 10:00 p.m., 43% of the population (mostly the young) headed to bed, while older children remained awake (p = 0.001). Having a mobile phone (odds ratio, OR = 2.5; p = 0.01) or tablet (OR = 2.5; p = 0.05) was a significant predictor of sleep issues in the logistic regression model.

Conclusion: The use of electronic media and the amount of time secondary school children spend on it can significantly alter sleep quality, through interrupted rest, and time, from a reduced duration of sleep. Parents and care providers can help by creating awareness about the negative effects of using electronic media on sleep and health among children.

## Introduction

We spend up to one-third of our lives sleeping, thus our general "sleep health" is an important issue to consider throughout our lives [1]. Sleep is a crucial predictor of general health and well-being and getting a decent night's sleep is critical for a person's consistent good wellbeing, both physical and mental. The ideal amount of sleep that adolescents (10-19 years) need is 9 hours and 15 minutes, and that does not change from 10 to 17 years old [1]. However, children in secondary schools do not sleep this long. Sleeplessness among adolescents has been linked with various negative health outcomes, like obesity [2], mood disorders [3], and psychological distress [4]. Besides, sleep deprivation has also been linked with difficulties in performing daily activities [5] and school work, which negatively impacts their performance [6]. Sleep deprivation has also been associated with difficulties in controlling emotions and aggression that can lead to violence [7]. The recent advancements and developments in the science and technology sectors have made electronic gadgets like tablets, smartphones, laptops, light emitting diodes (LEDs), and video game consoles easily available [8]. Among other reasons, the over-use of electronic gadgets has been significantly associated with sleeplessness as adolescents spend most of their evening time watching or using electronic media. It is reported [9] that about 80% of children 0-18 years old watch television before going to sleep. Adolescents (10-19 years) today are growing up immersed in digital media, which has both beneficial and negative implications for their healthy development [10]. This study is the first of its kind in Saudi Arabia to assess the quality of sleep due to the use of electronic media as the use has significantly increased among adolescents, which can have a detrimental effect on their sleep quality.

## Materials and methods

This was a cross-sectional study conducted from July 2021 to December 2021, in the Academy of Family Medicine, Madinah Al-Munawarah, which is located in northern Saudi Arabia. It covers an area of 151,990 square kilometers and has a population of 2,080,000 people [9]. There are 120 female secondary schools in Al-Madinah, Saudi Arabia, and this study examined eight female secondary schools using a multi-stage sampling technique. According to the WHO sample size calculator, the sample size was 375. Female students from a government secondary school in Al-Madinah participated in the study. The sampling technique used was the stratification of governmental schools according to their location in the north, south, east, and west. Then, using basic random selection, two schools were chosen. After obtaining their consent, 60 students from each chosen school were included in the study by cluster sampling. The research included all female students, but excluded those with chronic conditions, diabetes, hypertension, known sleep inducers, known antidepressant users, and a female known case of sleep apnea. In Arabic, a modified valid questionnaire was utilized to examine sleep patterns. The amount of time spent on screens during the day and on electronic devices before bed was monitored as part of the research. Madinah's regional research and ethics committee provided ethical approval. Confidentiality of data was maintained throughout the project.

Data were analyzed using the Statistical Package for the Social Sciences version 26 (SPSS Incl., Chicago, IL). Qualitative variables were presented as frequency and percent. Quantitative variables were tested for normality distribution and were presented as mean and standard deviation. The Chi-square test was used for group comparison. This study considered variables statistically significant at p < 0.05.

## Results

In this cross-sectional study, a total of 388 female students participated, from the age group of 12 to 40 years, with a mean age group of 16.45 ± 4.25 years. The majority of the students were in the age group 20 years (376/96.9%). Most of the students were in the third secondary (46.4%), followed by 131 (33.8%) in the second secondary, and 77 (19.8%) in the first secondary. Most of the participants were single (367/ 94.6%), while 13 were married, and 7 were widowed. Table 1 shows the demographic characteristics of the participants.

**Table 1:** Demographic data of the participants (n=388).

Variables	Frequency	Percentage
Age group		
<20 years	376	96.9%
>20 years	12	3.1%
Academic degree		
1^st^ secondary	77	19.8%
2^nd^ secondary	131	33.8%
3^rd^ secondary	180	46.4%
Marital status		
Single	367	94.6%
Married	13	3.4%
widower	7	1.8%
Number of children		
Nil	281	98.2%
<2	2	0.6%
>2	4	1.1%
Part-time Job		
No	366	94.4%
yes	22	5.7%
Medical diagnosed condition		
Yes	38	9.8%
No	350	91.2%

In this study, 215 participants used some sort of stimulant, like energy drinks and coffee. However, the rest were not using any stimulants. Before going to bed, most of the participants in our study used different kinds of electronic devices. Of those, 335 participants used electronic devices daily while going to school. However, the same question was asked regarding weekdays and 357 answered yes that they use electronic devices before going to sleep on weekends. When asked regarding the type of device used, most of the participants used their smartphones (85%), tablets were used by 6%, playstations and computers were used by 3% each, and only 1% used television. Sleep pattern characteristics are shown in Table 2.

**Table 2:** Sleep pattern characteristics of the participants (n=388).

Question	Less than a week	Not in the last month	Once or twice a week	Three or more times a week
During the last month, how many times did you have trouble sleeping				
Because you cannot be asleep in 30 minutes.	109(28.09%)	114(29.4%)	64(16.5%)	101(26.0%)
Waking up in the middle of the night or early morning	115(29.63%)	96(24.7%)	82(21.1%)	95(24.5%)
I have to get up and go to bathroom	133(34.27%)	137(35.3%)	79(20.4%)	39(10.1%)
Cannot breathe comfortably	111(28.60%)	211(54.4%)	40(10.3%)	26(6.7%)
Coughing or loud snoring	78(20.1%)	302(77.8%)	4(1%)	4(1%)
Feeling very cold	183(47.2%)	132(34.0%)	73(18.8%)	00
Had bad dream	149(38.4%)	137(35.3%)	47(12.1%)	55(14.2%)
Feeling bad	121(31.2%)	182(46.9%)	36(9.3%)	49(12.6%)
How many times medication was used for sleep?	90(23.2%)	288(74.2%)	00	10(2.6%)
Trouble staying awake while driving, eating, or during social activities	126(32.5%)	198(51.0%)	43(11.1%)	21(5.4%)

In Table 3 sleep pattern of the participants has been described. 

**Table 3:** Sleep pattern of the participants in our study.

Question	Less than a week	Not in the last month	Once or twice a week	Three or more times a week
If you have a bed partner or share a room ask him/her in the past month, how many times have you:				
snored loudly	153(39.4%)	198(51%)	19(4.9%)	18(4.6%)
A loud pause between breath during sleep	147(37.9%)	228(58.8%)	00	13(3.4%)
A man who is not calm during sleep	147(37.9%)	197(50.8%)	11(2.8%)	33(8.5%)
Bouts of sleep confusion	152(39.2%)	203(52.3%)	16(4.1%)	17(4.4%)

By doing a comparative analysis of electronic device use and sleep patterns, it was discovered that the more time spent on devices, the more disrupted sleep was (p-value = 0.005). One-third of third- and second-year students reported having trouble waking up in the morning. This issue was more prevalent in older children: 55% of children in their third year and 41% of children in their second year (p = 0.01). Over 20% had difficulties falling asleep. Fewer than 5% of respondents reported frequent nightly awakenings, whereas 23% reported being drowsy/sleepy during work. After 10:00 p.m., 43% of the population, primarily the young (p = 0.001), retired to bed. In the logistic regression model, having a cell phone (odds ratio, OR = 2.5; p = 0.01) or tablet (OR = 2.5; p = 0.05) was a significant predictor of sleep problems.

## Discussion

The aim of the study was to determine the association between electronic media use and changes in sleep patterns and sleep disturbances among adolescents. A significant association was found between the use of electronic media and disturbances in sleeping patterns such as weariness, restless sleeping, difficulty waking, and sleep quality. However, when course credits or school performance were added to the model, the effect of electronic media was significantly reduced, as sleep quality is also significantly associated with the course load. The use of electronic entertainment and communication devices (EECDS) was reduced with students’ enrolment in a full semester or more credit hours, as the school workload may not provide time for using electronic media and they mainly used it for working out their assignments. It was determined that in such students, the study burden was more responsible for sleep disturbances than the use of electronic media. The exciting content of the electronic media, including TV, video games, sports, etc., can keep children awake till late at night. Similarly, light or the noise that electronic media emits can also significantly reduce sleep time and quality as children tend to go to bed later due to arousal [11]. These results have also been verified by adolescents who have verified these results themselves that electronic media can cause sleep disturbances [12]. Melanin is a hormone that stimulates sleep and is secreted in greater quantities later at night and has been found to be reduced by light caused by electronic gadgets [13]. Development stages are also associated with changes in sensitivity to light, and adolescents are more sensitive to light than adults and non-puberty adolescents [14]. The most encouraging finding was a positive association between electronic activity and sleep/wake disruptions, implying that the more electronic activity a person reported, the more unsatisfactory sleep they experienced. Greater difficulty waking in the mornings was also predicted by increased use of electronic media. The proportional relevance of various technologies is still up for debate, while social communication devices have been suggested to have a particularly harmful impact on sleep [15]. However, there were few statistically significant differences across the electronic gadgets in the current investigation. Furthermore, because most platforms are multitasking and multipurpose (e.g., schoolwork versus leisure usage), caution is required in drawing conclusions about the association of sleep quality with electronic media usage [16]. Another objective of the study was to see whether types of electronic media were linked to poor sleep quality. The findings revealed a substantial link between watching TV less than 2 h before bedtime and sleep and wake disruptions. This is significant given that teenage TV viewing time averages 72.9 min [17] per day and that adolescent TV viewing indicates a greater risk of sleep disorders in adulthood [9]. In comparison to earlier research on teens, the current findings come from a largely college-based population. This suggests that late-night computer usage and sleep-wake disorders are becoming more common among college students. Similarly, computer use was linked to sleep quality. Adolescents spend an average of 56 min each day on computers [18-19]. The responses to weariness, sleeping and waking issues, and liking school are all consistent with physiological and psychological development aspects. At school, nearly all of the children aged six and seven seemed awake, with just a handful of them expressing that they were frequently weary (2.5%). Almost one-fifth (18.5%) of prepubescent children aged 10 reported feeling tired at school on a regular basis. Finally, in the pubertal period, more than 40% of teenagers aged 14 and 16 reported being weary frequently at school. Most of the teenagers had trouble waking up in the morning. This is in line with past findings, indicating that even it is less dominating in adolescence.

## Conclusions

Our study concludes that the use of electronic media and the amount of time secondary school children spend on it can significantly alter both sleep quality and time. Parents and care providers can help by creating awareness about the negative effects of using electronic media on sleep and health among children.
